# Automated image analysis with the potential for process quality control applications in stem cell maintenance and differentiation

**DOI:** 10.1002/btpr.2199

**Published:** 2015-11-28

**Authors:** David Smith, Katie Glen, Robert Thomas

**Affiliations:** ^1^ Centre for Biological Engineering, Wolfson School of Mechanical and Manufacturing EngineeringLoughborough UniversityLoughboroughLE11 3TUU.K

**Keywords:** pluripotent stem cell, image analysis, cell‐IQ, process control, automation

## Abstract

The translation of laboratory processes into scaled production systems suitable for manufacture is a significant challenge for cell based therapies; in particular there is a lack of analytical methods that are informative and efficient for process control. Here the potential of image analysis as one part of the solution to this issue is explored, using pluripotent stem cell colonies as a valuable and challenging exemplar. The Cell‐IQ live cell imaging platform was used to build image libraries of morphological culture attributes such as colony “edge,” “core periphery” or “core” cells. Conventional biomarkers, such as Oct3/4, Nanog, and Sox‐2, were shown to correspond to specific morphologies using immunostaining and flow cytometry techniques. Quantitative monitoring of these morphological attributes in‐process using the reference image libraries showed rapid sensitivity to changes induced by different media exchange regimes or the addition of mesoderm lineage inducing cytokine BMP4. The imaging sample size to precision relationship was defined for each morphological attribute to show that this sensitivity could be achieved with a relatively low imaging sample. Further, the morphological state of single colonies could be correlated to individual colony outcomes; smaller colonies were identified as optimum for homogenous early mesoderm differentiation, while larger colonies maintained a morphologically pluripotent core. Finally, we show the potential of the same image libraries to assess cell number in culture with accuracy comparable to sacrificial digestion and counting. The data supports a potentially powerful role for quantitative image analysis in the setting of in‐process specifications, and also for screening the effects of process actions during development, which is highly complementary to current analysis in optimization and manufacture. © 2015 The Authors Biotechnology Progress published by Wiley Periodicals, Inc. on behalf of American Institute of Chemical Engineers, 32:215–223, 2016

## Introduction

Therapeutic cell‐based products currently lack an established and mature set of compatible manufacturing technologies.^1^ Consequently, translating laboratory‐based processes into reproducible quality and economically viable commercial production techniques for market scale remains a challenge.^2^ Developing process integrated analytical systems and automated feedback loops that control product variation within acceptable limits is an important requirement to decrease production risks.

The development, qualification, and validation of analytical methods are important aspects of biological manufacture. The suitability of analytical tools for a process will determine the efficiency and precision of both process development and subsequent manufacture. Cell‐based therapies present some new challenges in process monitoring due to the complex and dynamic phenotypes of the cell populations and their sensitivity to the process environment; this currently indicates that a more comprehensive cell analytical solution will be required than that applied to cell lines producing macromolecule biologics. Although significant progress has been made to develop novel analytical tools for measurement of cells and their processing environment,^3^, ^4^, ^5^, ^6^, ^7^, ^8^ certain in‐process culture attributes are still not well understood or sufficiently controlled^9^, ^10^; this undetected variability may manifest in the cells throughout production, and transmit to the quality of the final product. Further development of Process Analytical Technologies (PAT) offers the potential for continuous quality assurance resulting in improved operational control and compliance, ultimately leading to more reliable product quality and lower production risks and costs.^11^

Cells prepared for therapeutic use need to maintain certain characteristics, such as viability and phenotype, to perform as required. The current analytical tools to assess this commonly use invasive sampling and sample processing to detect markers such as surface proteins or gene expression. This requires disruption of the process in the case of adherent cells, and potentially increases process operations and therefore process risk. It further requires sample sacrifice or careful assurance that clinical safety or efficacy is not altered by analytical techniques which could trigger cellular abnormalities, a particular issue for small scale high value processes.^12^ Noninvasive PAT, ideally automated, real‐time and continual, would reduce these issues.^13^, ^14^, ^15^ It may also reduce or remove post product analysis and reduce production failure through allowing reaction at the point of process deviation. This would have additional benefits for short shelf life products coupled with onerous post production testing time requirements.

Currently, user subjective visual inspections are frequently performed to inform process decisions e.g. an estimation of confluence to determine when to passage cells.^16^ Cell morphology is typically used to assess cell populations, with some ability to discriminate between phenotype based on shape and size.^17^ Currently there are no suitable technologies capable of accurately and reliably measuring such key critical attributes of cells in a manufacture process. Here we report the potential of an image analysis platform and software suite to undertake such analysis for the routine measurement and quantification of morphological culture attributes of pluripotent stem cells through phase contrast microscopy. We progress beyond previous studies to show precision of the methodology and sensitivity of morphological attributes to process operations; these are critical areas of understanding to enable application as a PAT.

## Methods

### Routine Maintenance of Cells

Human embryonic stem cell (ESC) line H9 (WiCell, Wisconsin) colonies were cultured on Matrigel (BD Biosciences, Oxford, UK) with mTeSR‐1 (Stem Cell Technologies, Vancouver) and passaged using Dispase (Stem Cell Technologies, Vancouver, Canada) following the manufacturers’ protocols.^18^, ^19^ Briefly cells were thawed and passaged three to four times prior to analysis, removing any differentiation at each passage using an aspirator. Six‐well plates (Fisher Scientific, Loughborough, UK) were coated with Matrigel 1 : 100 dilutions in cold DMEM (Fisher Scientific, Loughborough, UK) and refrigerated for up to one week prior to use as per manufacturer's instructions. Before use, the Matrigel coated flasks were removed from the fridge and left at room temperature to polymerize for at least 1 hour. The excess Matrigel solution was aspirated and warm mTeSR‐1 added. The cells were then added in solution with mTeSR‐1, rocked to evenly distribute, and placed in a 37°C/5%CO2 incubator. Media was replaced with fresh mTeSR‐1 every 24 h.

### Image Acquisition

The Cell‐IQ® (CM‐Technologies, Tampere, Finland) is a fully automated continuous live cell imaging and analysis platform, which has previously been described in detail.^20^ Briefly the Cell‐IQ houses an inverted microscope, a 4x objective (selected), CCD camera and an environmental chamber (37°C/5% CO2). Inside the chamber is a motorized 2 plate/flask stage. Phase contrast images were automatically captured every 30 min at pre‐programmed positions. Cells were removed for routine maintenance as above.

### Image Analysis

Image analysis was completed via the on‐board Cell‐IQ Analyser™ program (CM‐Technologies, Tampere, Finland). This program uses Machine Vision technology, a term given to the action of duplicating the effect of human vision by electronically perceiving and understanding an image.^21^ Here machine vision is utilized to identify populations of morphologically different cells based on “taught” pattern recognition and segmentation. A pluripotent analysis protocol was built using the Area Finder tool within Analyser. Briefly, this required libraries of example images of each colony morphology that are treated as distinct categories (see results). All captured images were compared to these libraries to categorize image areas into separate groups. Four areas were “taught” to the Cell‐IQ, background/debris, central core, peripheral core and edge cells. Analysis of in process images for classification involved utilising squares of 64 x 64 pixels. An algorithm then compares the captured images to the image libraries in order to classify areas into one of the four morphological categories. The pluripotent analysis protocol can be accessed here https://goo.gl/REZGS3.

### Immunostaining

Cells were fixed in 4% paraformaldehyde (Sigma Aldrich, Gillingham, UK) prior to blocking at room temperature for 30 min with Phosphate Buffer Solution (PBS) (Lonza, Cologne, Germany) containing 10% normal donkey serum (Sigma‐Aldrich) 0.3% Triton X‐100 (Sigma‐Aldrich, Gillingham, UK) and 1% Bovine Serum Albumin (BSA) (Sigma‐Aldrich, Gillingham, UK). After blocking, samples were incubated with their respective conjugated antibodies in blocking buffer for 3 hours at room temperature in the dark. Conjugated antibodies used were Sox‐2 (NL557, R&D Systems), Oct‐3/4 (NL637, R&D Systems), and HAND1 (NL637, R&D Systems). Following a wash step in PBS, cells were incubated with 5 µM SYTO‐16 or DAPI (Life Technologies, Paisley, UK) solution for 10 min. A final wash in PBS was completed before viewing the samples in PBS on a Nikon Ti Eclipse microscope.

### Flow Cytometry

Samples were fixed and permeabilized using Cytofix/Cytoperm (BD Biosciences, Oxford, UK) as per manufacturer's instructions. Then respective antibodies were added to the cells (Sox‐2 [AF647, BD Sciences], Oct‐3/4 [PerCP‐Cy5.5, BD Sciences], Nanog [PE, BD Sciences] and incubated for 1 h at room temperature. Flow cytometry was carried out using the InCyte software on Guava 8HT Cytometer (Millipore, Watford, UK), and analyzed using FlowJo Version 10.

### Differentiation

In order to induce early mesoderm differentiation in the embryonic colonies, the pluripotent mTeSR‐1 media was supplemented with BMP‐4, FGF, VEGF and Activin A all at 10 ng/ml (R&D Systems, Abingdon, UK). This supplemented media was replenished every 24 h until final analysis.

### Automated Cell Counting

Cell cultures were washed twice with room temperature phosphate buffer solution (PBS) (Lonza, Cologne, Germany). Accutase (Sigma Aldrich, Gillingham, UK) was prewarmed and added to each culture vessel before incubating at 37°C for 7 − 10 min. Accutase was quenched by added fresh mTeSR‐1 (Stem Cell Technologies, Vancouver) and counts were conducted in triplicate using Cedex (Roche Innovatis).

### Statistical Analysis

All statistical analysis including regression, ANOVA and statistical attributes were conducted using Minitab 16 software.

## Results

### Correlating Cell Morphology to Conventional Analysis

**Figure 1:**
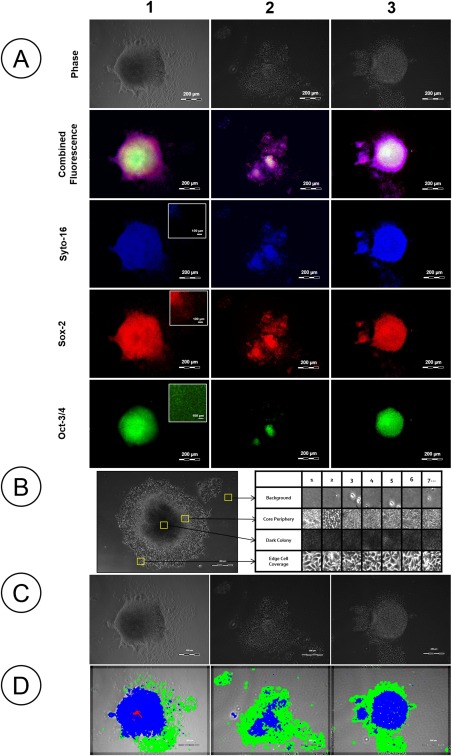
H9 embryonic stem cell colonies: morphology and relationship to key transcription factors. A: Immunostained images for Syto‐16 (nuclear stain), Sox‐2, and Oct‐3/4. Sox‐2 stains all cells, whereas Oct‐3/4 is more specific to the colony core. This identifies that colony areas are phenotypically distinct for key markers and that measuring the associated morphologies is potentially relevant to in‐process quality assessment. Inset images provide higher magnification to highlight the nuclear staining. B: Examples of the images used to build the reference libraries for classification of different morphologies: three distinctive colony regions (and background) are shown representing morphologically distinct populations. C: Three representative phase contrast images of embryonic colonies and (D) the output of the images after analysis using the Cell‐IQ Analyser™ software and reference libraries showing successful identification of distinct areas; dark central core colony (red), edge cell coverage (green), and periphery core (blue).

To determine the association between colony morphology and phenotype, and therefore justify a role for morphology in in‐process control, morphologically different hESC colony areas were assessed for grossly different phenotype by immunostaining for master regulators of pluripotency Oct3/4 and Sox‐2.^22^, ^23^ Discrete micro‐environmental areas within the colonies differentially expressed these key factors (Figure 1A). All cells within the population were positive for Sox‐2, however the Oct‐3/4 staining was absent in peripheral cells and discretely present in the colony core. This separation in phenotype provides a mechanistic rationale that sub‐division of the colony by morphology will have value for process monitoring.

In order to quantitatively measure in‐process morphology an image analysis software tool was developed for identification and quantification. The Cell‐IQ Analyser™ software, was used to create four libraries (200 images each) of three areas of categorically distinct morphology (Figure 1B). These libraries covered the two areas identified as distinct by immunostaining, and a further subdivision of the central colony area into central core and core periphery in an attempt to identify an anticipated transitional state. Use of these libraries to analyze a series of phase contrast colony images showed it was possible to reproducibly identify these target colony morphologies (Figures 1C,D show three representative examples). In general, the central core corresponds to a relatively dark and dense area of coverage (shown red), the edge cells are relatively low density and spread (shown green) and the band of core periphery are morphologically intermediate between the two (shown blue). The green and blue areas were selected for further analysis due to their respective approximation to the Oct‐3/4 negative and positive areas observed under immuno‐staining and their sensitivity to culture changes as described below.

### Morphology Sensitivity to Process Operations

The change of the different morphological characteristics were monitored over a set of different operational scenarios to provide evidence that the derived metrics are sensitive to important changes in the cell culture environment, a key test for utility as a PAT.

#### Image Analysis in Pluripotency Maintenance

**Figure 2:**
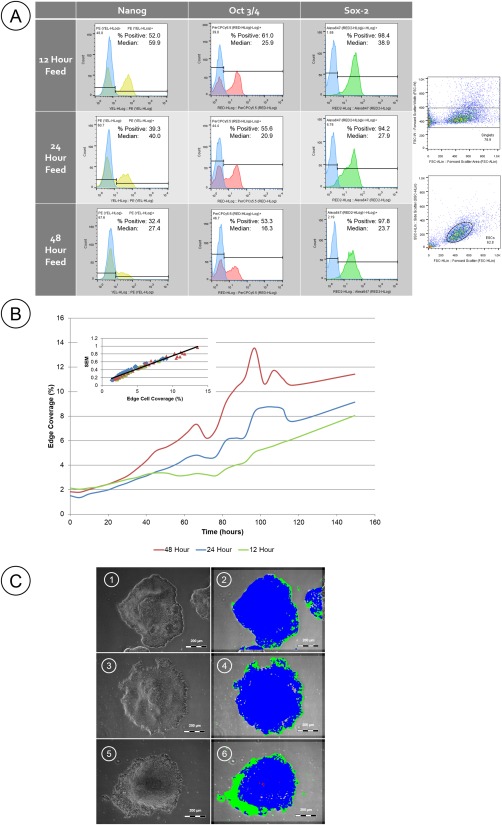
A: Flow cytometry analysis shows a reduction in pluripotent markers (Nanog, Oct‐3/4, and Sox‐2) with increased media exchange intervals (12, 24, 48 h; isotype shown in blue). Results taken from an average of 6 wells of each condition. Also shown are example dot plots showing the gating on initially “singlets” and then “ESCs,” prior to hisotgrams of each marker with its respective isotype control. B: Percentage of cell coverage categorized as “edge” using Cell‐IQ Analyser™ software. Analysis of phase contrast images shows increase in edge cell coverage with increased media exchange intervals [12 h (green), 24 h (blue), and 48 h (red)], *n* = 6. Inset image shows Standard Error of the Mean (SEM) of coverage dependent on level of coverage as an indication of precision. C: Representative phase contrast images (pre 1,3,5 and post 2,4,6 analysis) taken from 12 h 1, 2, 24 h 3, 4, and 48 h 5, 6 media exchange regimes, showing less frequently fed cultures had greater edge cell coverage.

Media exchange regime is a key determinant of cell phenotype, and hence cell product quality. Online monitoring that can detect deviations due to variable media quality or constituent decay rates will be valuable in manufacture. To identify the sensitivity of the image analysis tool to deviations in media supply three different media exchange regimes were implemented for maintenance of pluripotent stem cells over 5 days and the quality of the cells were monitored by flow cytometry and image analysis. Gating was completed initially to remove any doublets, followed by selecting the cell population, and finally gating off 95% off the isotype controls (Figure 2A). A reduction in all pluripotent markers (Nanog, Oct3/4 and Sox2) was measured as the media frequency replacement was reduced; in particular Nanog, where the median fell from 59.9 to 27.4 in the 12 hour and 48 hour media exchange regime respectively (Figure 2A). The image analysis showed a corresponding divergence in the level of “edge” cells observed in different feed regimes, with greater edge cell morphology appearing with less frequent media exchange, indicating that the analysis is sensitive to the changes induced in the cells (Figures 2B,C). This loss of pluripotent markers in conjunction with an increase in edge cell morphology is supportive of the initial immunostaining data showing lower expression of Oct3/4 in peripheral colony cells.

The precision of the methodology to quantitate edge cell coverage was determined using the standard error of the mean (SEM) for the 76 image sample for different levels of coverage (inset Figure 2B). The change in SEM is approximately proportional to the change in edge cell coverage indicating similar precision relative to the mean coverage value throughout the range. The SEM can also be used to determine the number of randomly sampled images required to detect a real morphological deviation between populations; sampling can therefore be tuned to sensitivity requirements, and maximum efficiency, for the application. For example, a 76 image data set used at 10% coverage gives a 95% confidence interval of 10 ± 1.5%. This indicates that a large number of culture vessels could be monitored with relatively low imaging and analytical demand, and that the method could be used for both screening the effect of process changes such as media interval, and for monitoring performance in subsequent manufacture.

#### Image Analysis in Differentiation

The observation that edge cell morphology to core cell morphology ratio is informative in measuring quality of cultures during pluripotent cell maintenance suggests the same morphological categories may identify the initial stages of directed colony differentiation. It is also plausible that a morphological specification could be used to determine a suitable initiation point for differentiation or a response point thereafter. This would be useful because the state of the colonies is widely recognized to be critical in determining early differentiation.

**Figure 3:**
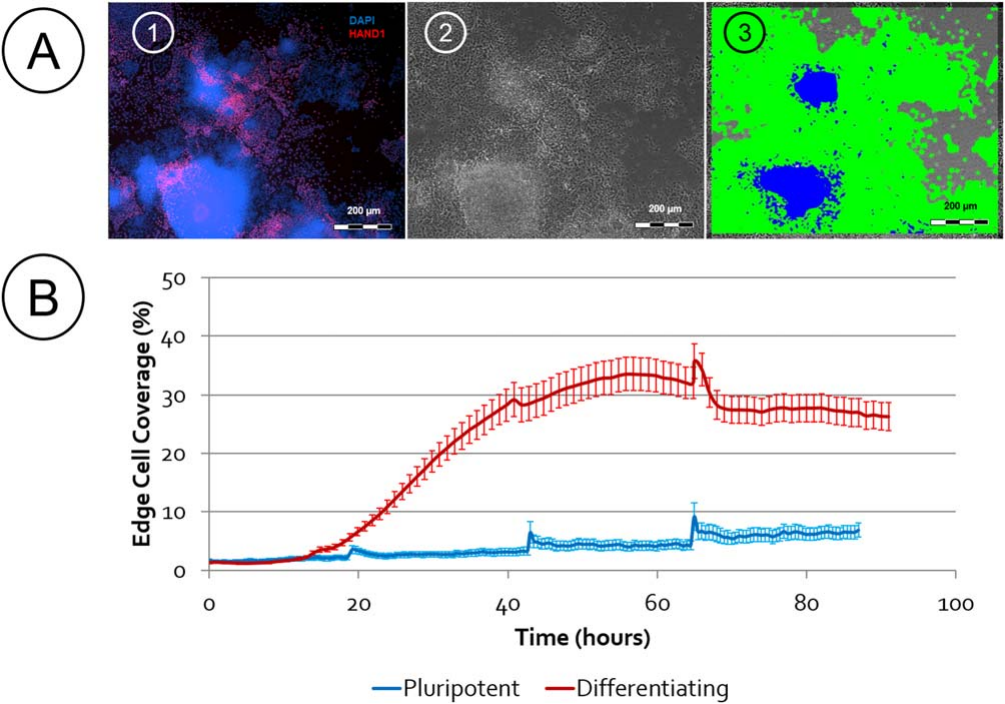
A: HAND1 expression (red) confirms early mesoderm commitment with DAPi counterstaining (blue). 1 Analysis of the phase contrast images 2 with analyzer and the three morphology libraries indicates the differentiating cells (predominantly HAND1 expressing areas) are categorized as edge cells, shown in green with the remaining colony core as blue 3. B: Quantitation of edge cells in cultures maintained in pluripotent support media compared to those that have received a differentiation stimulus shows a clear divide after 20 h (*p* ≤ 0.05). The peaks in coverage that occur in both pluripotent and differentiating populations every 24 h represent feed points. The images succeeding each feed point required refocusing giving a temporary anomaly (data based on 36 images, error bars represent SEM).

In order to explore this application, early mesoderm differentiation was stimulated (BMP‐4, VEGF, FGF, and Activin A) and the response of the colonies monitored. In order to report differentiation at the earliest time point early mesoderm commitment was confirmed through HAND1 expression^24^, ^25^ (Figure 3A). Morphologically, an outgrowth from the colony periphery was observed in response to the differentiation stimuli; this was classified using image analysis as edge cell coverage. After 15 h a separation can be quantified through image analysis between a culture exposed to a differentiation stimuli and a culture maintained in pluripotency media (Figure 3B). Statistical separation of the pluripotent and differentiating populations was achieved after 20 h using an image sample size of 36 (*p* ≤ 0.05), suggesting a very early quantitative indicator of protocol progress (relative to conventional marker analysis) can be gained with limited imaging and analysis.

The observed relationship between both morphology and pluripotent colony phenotype, and morphology and the colony differentiation response, in combination with the ability to analyze the development of multiple colonies individually, allows correlations to be sought between the morphological state of cell input colonies and the subsequent response to a process action. It is therefore possible to use the methods discussed as an optimization screening tool. This is potentially valuable to decrease irreproducibility of early differentiation processes that is in part due to variable colony states at the point of differentiation stimulus, and hence variable responses.

**Figure 4:**
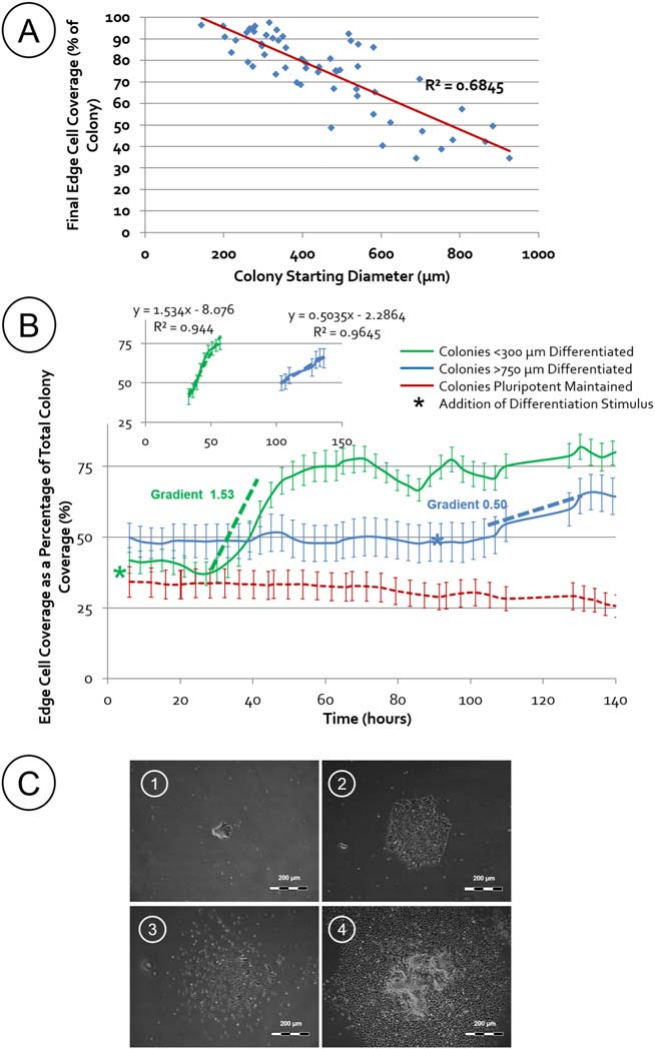
A: An analysis of the starting colony size with respect to the morphological response of the colony to mesoderm differentiation stimulus, as measured by percentage of edge cell coverage. This indicates that smaller colonies show a greater morphological response to a mesoderm differentiation stimulus where nearly 100% of the colony becomes morphologically “edge”. The larger colonies show less response and maintain a percentage of normal colony morphology. 54 images from 3 separate wells. B: A differentiation stimulus was added when a given mean colony size was reached, either <300 µm (green line) or >750 µm (blue line). Colonies remaining in pluripotent media without differentiation stimuli are shown as a control (red line). A sudden increase in edge cell coverage highlights the morphological change after addition of a differentiation stimulus (30 h for small colonies and 100 h for the larger colonies). Inset shows linear regression of the edge cell response to differentiation stimulus, calculating the gradient of colonies <300 µm (green) and >750 µm (blue). *n* = 24 colonies, error bars represent SEM (C) Example images show the difference in morphology based on starting size; where a smaller colony completely becomes edge cell (1 and 3), whereas the larger colony maintains a central core (2 and 4).

This application was demonstrated by determining the effect of colony size on the colony response to the differentiation protocol. A heterogeneous population of colonies of varying size were treated with the mesoderm differentiation cocktail and edge cell coverage was used as a quantitative surrogate for the differentiation response achieved within the population. An inverse correlation was observed between the size of a colony and the proportion of the colony converting to edge cell morphology (Figure 4A). Smaller colonies undergo a complete transition to edge cell morphology, whereas the larger colonies maintain a central core morphology, suggesting a greater and lesser degree of differentiation (and homogeneity) respectively. This indicates that when a differentiation protocol is applied the proportion of the population that will have reached an early committed state (similar to that expressing HAND1 in Figure 3A) after a given amount of time, will be highly dependent on the initial size distribution of the colonies. Further protocol steps would likely have different outcomes if applied to populations that had reached different states of maturity. This suggests that colony based differentiation protocols should specify size distributions (and morphology of colonies at input and in early steps) to improve control.

In order to demonstrate how these observations could be applied to process control, colonies were grown in a pluripotent state with continual monitoring and the cytokine cocktail differentiation stimulus added when mean colony diameter had reached 300 µm or 750 µm. The morphological change that occurred in response to the differentiation stimuli was different depending on the colony starting sizes. The rate of change in edge cell morphology was faster in the smaller diameter colonies compared to the colonies with larger diameters (Figure 4C). This is consistent with the outcome from the prior experiment. As seen previously there is a residual colony core remaining after differentiating larger colonies (Figure 4B). This core prevents edge cell coverage for larger colonies reaching above 70%. For the smaller colonies, more complete morphological differentiation occurs, with up to 86% edge cell coverage.

### Sampling for Control

A key benefit of noninvasive image analysis is that it is nondestructive. This enables more extensive sampling and therefore potentially increased precision. This is important in cell therapy processing where many candidate manufacturing processes work with multiple small scaled‐out units such as cell culture flasks; sampling individual units can be poorly predictive of whole process performance due to high unit to unit variability.

Although the precision of the morphology measurement can be assessed (and is reported above) through SEM for various coverage and sample rates, a precision comparison with conventional invasive methods is not directly relevant as conventional invasive methods are measuring different attributes; in essence the morphology is proposed as a new quality indicator with immunostaining and flow cytometry data presented in support of relevance. Cell count, however, is a commonly used process control measurement, with a direct comparison available. We therefore determined the potential of the image analysis tool to non‐invasively quantify cell number in a flask.

**Figure 5:**
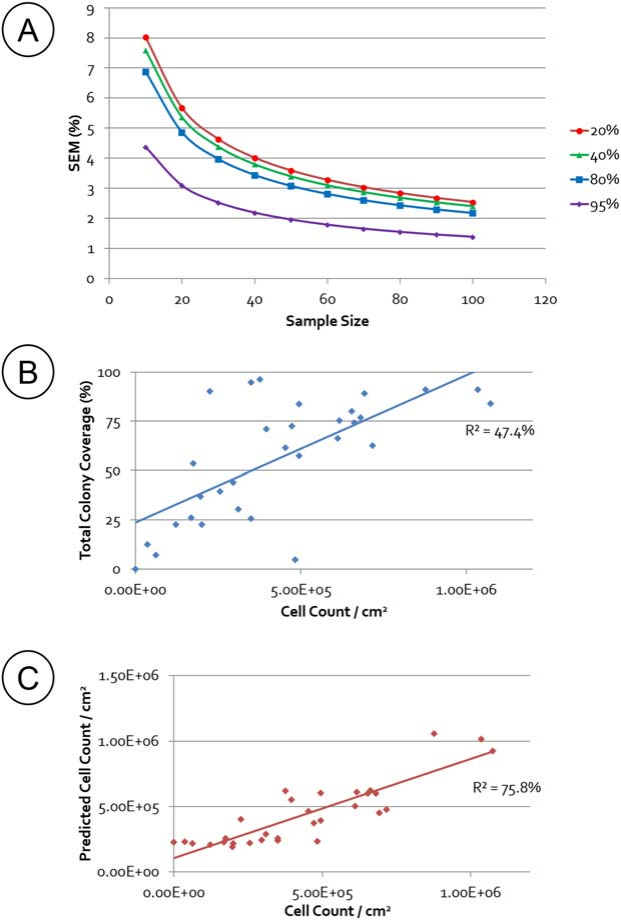
A: Relative standard error of the mean (SEM) against sample size for 400 images from 4 flasks of varying coverage values. This can be used to guide the sample size needed for the required precision. *n* =31. B: Correlation of image analysis coverage to a cell count using the sample rate of 40, which gave an SEM of <4% at all coverage levels‐produced an *R* ^2^ of 47.4%. C: In order to account for the various densities within colony culture the three colony morphology libraries were used to quantify different morphological areas. Using multiple linear regressions a better correlation of image analysis metrics to cell count was completed, with a *R* ^2^ of 75.8%. *n* = 3.1.

400 random images were acquired for a range of different cell densities. The SEM of this data set was used to determine the sample size needed to give less than a 4% SEM in coverage precision across the confluence range (Figure 5A). Presuming that each cell was the same size and nonoverlapping, this calculated image sample rate of 40 would provide a good correlation with cell number counted by culture digestion; however, the correlation was relatively poor (Figure 5B, *R* ^2^ = 47%). In an attempt to account for coverage areas that were of higher or lower density, multiple linear regression was conducted using coverage of the three distinct colony areas identified above. This provided a better correlation with counted cell number (Figure 5C, *R* ^2^ = 76%); the coefficients of the regression equation show that the central core, followed by the core periphery have far greater weighting in predicting total cell number; a logical consequence of these areas containing greater cell numbers. This is distinct from the previous work monitoring quality in maintenance and differentiation where the central red area was not relevant due to lack of change across experimental ranges.

Sacrificing a single unit to assess the state of a culture introduces error based on the representativeness of the sampled unit. Distribution of cell yields were analyzed from 10 flasks in a single batch and found to have a coefficient of variation (CV) of 0.26, suggesting a 95% confidence interval of ±15% for predicting batch yield from a single flask. On the contrary, the image analysis described can provide a highly precise assessment of coverage; although in this case the faithfulness of the correlation to cell number will introduce error. However, this error will be random from flask to flask and therefore quickly reduce with assessment of multiple units. In some instances, image analysis of multiple units is therefore likely to provide a competitive or superior assessment of cell number in culture than a sacrificial unit.

## Discussion

We have developed and applied a process analytical technology (PAT) for the automated quality assessment of pluripotent colonies that is precise, rapid and noninvasive. Phase contrast images of label free cultures can be analyzed to provide quantitative data that meaningfully relates to known quality markers and can be used to make process decisions and assess the effect of process actions. The level of imaging required to provide statistical discrimination between different process states is relatively low suggesting the methodology could be implemented to monitor human pluripotent cultures to provide feedback control for in‐process decisions.

Current monitoring tools are not suitable for in‐process control of cell therapy production.^13^, ^20^ These cultures are highly complex and heterogeneous relative to cell lines used in conventional biopharma; the major challenge is to identify relevant sets of quality attributes and associated analytical tools that provide sufficient quantitative insight into cell behavior during manufacture to enable process decisions.^26^, ^27^

It is unlikely, in the near future, that knowledge and methods will be available to directly quantify all parameters that causally determine the response to any process action for systems as complex as hESC culture. The number of potential single biological markers that could be measured, such as proteins or mRNAs, and their distribution across the cell population, are daunting for selecting an informative but practicably small monitoring panel. The complexity and the sensitivity of the culture systems demand extensive analysis for control, but the cost and material limitations paradoxically demand limited monitoring panels. Further, the more limited the range of cell attributes measured, the more likely that they will not be predictive of cell behavior over the range of operating scenarios required.

Although quantitative morphology assessment does not wholly overcome these issues, it is a single measurement that will be representative of a large number of underlying biological factors. It therefore provides a broad assessment that may respond to a range of underlying single factor deviations, not all of which may be amenable to monitoring independently or even known. Furthermore, as biological systems are complex networks, single factor changes are unlikely to occur in isolation, suggesting that for process monitoring and control purposes greater efficiency could be achieved by measuring crude aggregate response to system change (such as morphology) rather than individual factors. Furthermore, the nondestructive nature of the measurement opens the opportunity for continual or high frequency analysis and high sampling rates; this both increases precision (tailored to requirements) and allows rates of change to be considered alongside absolute values. This provides a powerful rationale for the inclusion of quantitative morphology data alongside single factor analysis in process control. Although the functional significance of morphology is less easy to mechanistically explain compared to biological markers that form parts of known networks, accumulating experience and data will inform normal statistical ranges for these properties and allow setting of operational brackets for the PAT. It is important for application in PAT that the precision of the morphology measurement is defined, and that the morphology is shown to be sensitive to important process changes. It is not necessary to quantify a direct correlation to known markers unless the method is intended as a direct surrogate for those markers.

The value of morphology data is dependent on the image library used to generate the analysis algorithm. This work utilized library sizes of 200 for each morphological category. This was a trade‐off between both precision and time. Increasing the library size further had limited effect on precision (evaluated by assessing misclassified events) but impacted by increasing the protocol building time, a drawback for real‐time monitoring. This approach could have maximum impact if standardized and shared libraries were available for analysis, similar to reference standards in other analytical fields. Libraries could then be evolved with optimized sensitivity to detect specific features, for specific lines, and to assess comparability between sites. Such an approach would lead to subsets of libraries for different process requirements; for example we have reported that of the three morphological categories identified in this work, two were sensitive to tracking changes to cell quality in maintenance and differentiation, and an alternate pairing was better for tracking cell numbers. Such categorizations will be relatively specific to a given culture format and possibly even cell lines; an increase in the application of image analysis and data pooling is required to establish how broadly applicable individual analysis algorithms will be, or whether individual lines and processes will require individual analysis algorithms to provide sufficient sensitivity.

The use of morphology for process control initially requires only known statistical ranges and some awareness of a relationship between morphology and required function. The use of morphology for optimization requires potentially greater knowledge of functional effect i.e. not just that changed morphology is linked to a deviation from desired function, but the specific morphology‐function relationship. We have begun to explore this through the correlation of cell counts to these image derived metrics and with the identification of colony size and consequent response to a differentiation stimulus; however for specific applications and differentiation protocols there is an extensive level of experimentation that could be conducted to determine the various morphological states that best predispose to different lineage commitment with low variability.

## Conclusions

Quantitative image analysis can form a powerful process development and control tool for pluripotent cell based products. It fulfils many of the key desirable features of PAT: informative, noninvasive, real time, and relatively continuous. The different characteristics discussed relative to conventional quality assessment make it highly complementary to current tools and suggest applications in optimization and manufacture. Increased availability of advanced image analysis platforms will warrant consideration of standards for comparability to maximize the value to the scientific community and product developers.
